# Anthrarobin

**Published:** 1888-06

**Authors:** 


### ANTHRAROBIN.

It is to Liebermann that dermatologists owe the introduction of this new drug, the chemical composition of which is much like that of chrysarobin. It is a powder, with large grains, whitish yellow, and when placed in contact with the nasal mucous membrane, causes a special irritation. It may be mixed with oil, or incorporated with lard or lanolin. It dissolves in 6 parts of glycerine (at a temperature of about 100° C. ?), as well as in a mixture of 10 parts of cold glycerine, and 5 parts of boiling alcohol, when it takes a deep yellowish-brown color. It dissolves in the same proportion in an aqueous solution of borax; in pure water it is but very slightly soluble.

G. Behrend has used this drug in all the dermatoses in which chrysarobin is used. The following are some of the formulas used:

*Pomades:*

| | |
|---|---|
| Anthrarobin | 10 grams. |
| Olive oil | 30 " |
| Lanolin | 60 " |

| | |
|---|---|
| Anthrarobin | 20 grams. |
| Olive oil | |
| Lanolin aa | 40 grams. |

| | |
|---|---|
| Anthrarobin | 10 grams. |
| Olive oil | 15 " |
| Axungium | 75 " |

| | |
|---|---|
| Anthrarobin | |
| Olive oil aa | 20 grams. |
| Axungium | 60 " |

*Alcoholic Tincture:*

| | | |
|---|---|---|
| Anthrarobin | 10 | grams. |
| Alcohol | 90 | " |

Dissolve.

| | | |
|---|---|---|
| Anthrarobin | 20 | grams. |
| Alcohol | 80 | " |

Dissolve the anthrarobin in the boiling alcohol.

*Glycerine Solution:*

| | | |
|---|---|---|
| Anthrarobin | 10 | grams. |
| Glycerine | 80 | " |

Dissolve by heat.

*Aqueous Solution of Anthrarobin and Borax:*

| | | |
|---|---|---|
| Anthrarobin | 10 | grams. |
| Borax | 8 | " |
| Distilled water | 80 | " |

| | | |
|---|---|---|
| Anthrarobin | 20 | grams. |
| Borax | 35 | " |
| Glycerine | | |
| Alcohol aa | 90 | " |

Behrend has used these formulæ in a large number of cases. He has continued the external use of the drug for weeks without causing pruritus or inflammation of the skin, even when applied to the face and to the eyelids. Immediately after frictions with it there is a certain amount of burning, but this disappears after a few minutes, as a rule, though it may last for an hour.

In regard to its therapeutic action, anthrarobin holds a place between chrysarobin and pyrogallic acid, but it has the advantage over the latter of having no toxic properties. Its action is markedly increased by previous frictions of the skin with black soap, or with spirit of soap. When applied to the skin it causes a brownish color, indicating oxydation, and it is to this phenomenon that its therapeutic action is probably due.

Up to the present time Behrend has used the drug in psoriasis, herpes tonsurans, and erythrasma, and has reason to be well satisfied with the results.—*Therapeutische Monatshefte*, No. 3, 1888.

